# Clinicoradiological Characteristics of Gastric Metastases: A Single Center Retrospective Study

**DOI:** 10.7759/cureus.30825

**Published:** 2022-10-29

**Authors:** Shota Tanaka, Rika Yoshida, Takeshi Yoshizako, Hajime Kitagaki

**Affiliations:** 1 Radiology, Shimane University Faculty of Medicine, Shimane, JPN; 2 Radiology, Shimane University Faculty of Medicine, Izumo, JPN

**Keywords:** abdominal radiology, computed tomography, oncology, stomach, gastric metastasis

## Abstract

Background: With recent advances in treatment, gastric metastases are increasingly becoming the subject of diagnostic imaging. On the other hand, it is difficult to detect gastric metastasis on CT finding images.

Purpose: To characterize the CT findings of gastric metastasis and investigate its treatment method and natural history in patients.

Materials and Methods: We retrospectively reviewed the CT findings of 15 patients diagnosed with gastric metastasis between April 2003 and December 2019 in our hospital. The location, size, and shape of the tumors on CT were evaluated. Moreover, their medical records, characteristics, complications, treatment options, and natural course were evaluated.

Results: Of the 15 patients with gastric metastasis, 9 were male and 6 were female. The median age was 74 (55-87) years. Gastric metastasis was diagnosed simultaneously with primary cancer in five patients. In other patients, the median interval time from the date of primary cancer diagnosis to gastric metastasis diagnosis was 27 (10-259) months. CT findings revealed that the gastric metastasis had a median size of 18 (12-135) mm and was mainly located in the middle third of the stomach. In addition, most patients had a submucosal tumor (SMT) pattern, followed by diffuse wall thickness and polypoid patterns (11 [73.3%], 3 [20.0%], and 1 [6.7%], respectively). The median time to death after gastric metastasis diagnosis was 112 (17-883) days.

Conclusion: The SMT pattern in the middle third of the stomach is the characteristic CT finding of gastric metastasis.

## Introduction

Malignant tumors rarely metastasize to the stomach, accounting for only 2.3%-5.9% of autopsy cases [1-2]. Several cancers, such as malignant melanoma, lung cancer, esophageal cancer, and breast cancer, metastasize to various organs but rarely to the stomach in daily clinical practice [3].

With the recent development of treatment methods including chemotherapy, the number of long-term survivors of cancer with distant metastases is increasing, and the chances of gastric metastases being targeted for diagnostic imaging is increasing. On the other hand, symptoms of gastric metastasis are vague, and it is difficult to identify gastric metastasis on CT findings images. Early detection of gastric metastasis can lead to early treatment and improvement of quality of life. CT exam is widely utilized for surveillance of many types of malignancies and endoscopy is not usually performed except for cases with gastrointestinal manifestation. Therefore, CT is the initial modality to describe gastric metastasis. Therefore, it is important to organize CT imaging findings of gastric metastases. However, imaging findings of gastric metastasis have only been reported in case reports and need to be studied in a coherent number of cases [4-6].

The purpose of this study is to characterize the CT findings of gastric metastasis and to investigate the treatment and natural history of this condition in patients.

## Materials and methods

Our institutional review board approved this retrospective study and waived the requirement of informed consent from each patient. This study retrospectively reviewed the hospital’s information system and radiological information system and found 15 patients diagnosed with gastric metastasis between April 2003 and December 2019. Their medical records, characteristics, complications, treatment options, and natural course were evaluated

The inclusion criteria were as follows: 1) age over 20 years, 2) a history of any cancer, and 3) a CT-detected gastric tumor. The exclusion criteria were the following: 1) a history of gastric cancer; 2) a history of gastric surgery such as total gastrectomy, distal gastrectomy, or proximal gastrectomy; 3) cases of direct invasion of cancer into the stomach; and 4) oral contrast-enhanced media in the stomach.

CT technique

Abdominal CT was performed using various scanners, such as the following: GE CT9800 (GE Healthcare America, WI, USA), SIEMENS SOMATOM PLUS40 (Siemens Medical Solutions, Forchheim, Germany), PHILIPS Brilliance CT40, PHILIPS Brilliance CT64 (Philips Healthcare, Netherlands), TOSHIBA Aquilion 16, TOSHIBA Aquilion ONE, TOSHIBA Aquilion CX (Toshiba, Tokyo, Japan), and Canon Aquilion ONE (Canon Medical, Tokyo, Japan).

For contrast-enhanced CT, 100-150 mL of iodinated contrast material was administered at 2-5 mL/s and scanning was performed with a bolus-tracking technique [7].

An oral contrast medium was not used routinely. Whole-abdomen images were produced, showing varying section thickness depending on the period it was photographed: 10 mm, 2003-2004; 7 mm and 5 mm, 2006 onward. Abdominal CT images and soft-tissue window images were reconstructed by obtaining three cross-sectional images (namely, axial, coronal, and sagittal images; width, 350 HU; level, 30 HU).

Image interpretation

Two radiologists（S.T. and R.Y.）who had 14 and three years of experience, respectively, assessed the abdominal CT images independently using the Digital Imaging and Communications in Medicine (DICOM) viewer (SDS DICOM Viewer; Techmatrix Ltd., Tokyo, Japan). Both were blinded to patients’ clinical information but were aware that each patient had gastric metastasis. They evaluated the following points: 

1) Clinical findings of gastric metastasis

The date of the CT scan when gastric metastasis was first diagnosed was reviewed.

2) CT findings of gastric metastasis

**Figure 1:**
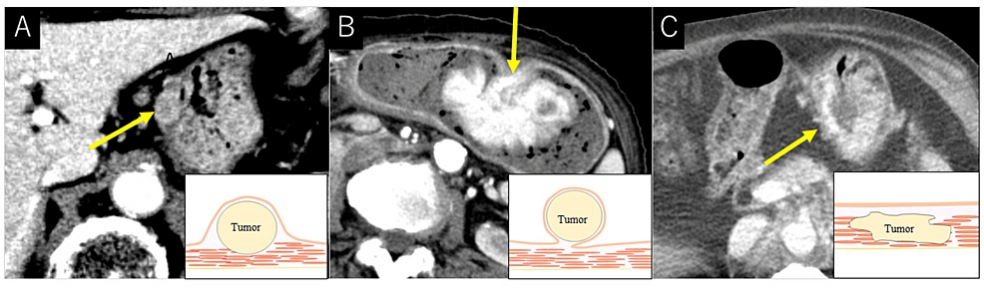
CT findings of metastatic gastric tumor with schema. A, SMT pattern; B, polypoid pattern; C, diffuse pattern SMT, submucosal tumor

Two radiologists independently reviewed the abdominal CT images and determined the location, shape, and size of the tumor. The location was decided by dividing the stomach into three parts: upper third, middle third, and lower third. The shape was classified into the submucosal tumor (SMT) pattern, polyp or polypoid pattern, or diffuse wall thickness pattern [1-3] (Figure 1). For the tumor size, we measured the longest diameter on CT. This study defined SMT as a gently rising, superficially smooth, localized elevated lesion. A polypoid lesion referred to a pedunculated elevated lesion. Wall thickening without the normal layered structure of the gastric wall, where the boundaries of lesions are vague, indicated a diffuse wall thickness pattern. The contrast pattern of the tumor was evaluated in three patterns (hypovascular, moderate, and hypervascular) in comparison with normal liver parenchyma in the equilibrium phase, and the results were favorable for hypovascular, moderate, and hypervascular tumors.

The two radiologists independently evaluated the CT findings according to the given definitions. Disagreements were resolved through consensus. The size was decided by averaging the measurements made by these radiologists.

Treatment options and clinical outcomes during follow-up

The hospital information system was retrospectively reviewed to evaluate the following aspects of the patient's treatment options:

1) Clinical symptoms associated with gastric metastasis

2) Treatment options

After assessing the gastric metastasis diagnosis, we investigated the treatment options in each patient by analyzing the medical records. These options were conservative treatments such as best supportive care (BSC) and chemotherapy, and interventions such as endoscopic resection and surgery.

3) Clinical outcomes

Patient death or survival at the last follow-up was considered. In the case of death, the number of days from the date of diagnosis of gastric metastases to the date of death was counted.

## Results

Patients

The study patients consisted of nine males and six females, with a median age of 74 (55-87) years at diagnosis. Table 1 shows patients’ information, background, and clinical findings. The pathological diagnosis of gastric metastasis was confirmed in 10 patients. Although pathological proof was not obtained in five cases, they were included in this study because they were clinically determined to be gastric metastases.

**Table 1:** Patient and clinical information. *Note*: M, male; F, female; melanoma, malignant melanoma; DLBCL, diffuse large B cell lymphoma; clear, clear cell carcinoma; adeno, adenocarcinoma; N/A, not available; Li, liver; Pe, peritoneum; Lu, lung; LN, lymph node; Ad, adrenal gland; Bo, bone; Pl, pleura; Pa, pancreas; SI, small intestine; Br, brain; S, skin; BSC, best supportive care; ESD, endoscopic submucosal dissection; chemo, chemotherapy

No.	Age/Sex	Primary cancer site	Initial stage (UICC 10th)	Interval from primary cancer diagnosis (days)	Symptom	Pathology of gastric tumor	Metastasis of other parts at diagnosis of gastric tumor	Treatment for gastric tumor	Interval from diagnosis to death (days)
1	77 M	Colon	IV	1250	None	N/A	Li, Pe, SI, LN	Chemo	N/A
2	73 M	Colon	IV	Simultaneously	None	Adeno	Li, Pe, LN	Chemo	758
3	67 M	Colon	II	7768	None	Adeno	None	Surgery	N/A
4	65 M	Lung	IV	Simultaneously	Pain	Adeno	Lu, SI, LN	ESD	79
5	70 M	Lung	IV	287	Pain	SCC	Lu, Br, LN	BSC	51
6	55 M	Pancreas	IV	570	None	Adeno	None	Surgery	749
7	74 M	Esophagus	IV B	Simultaneously	None	SCC	Lu, LN	Surgery	N/A
8	74 F	Ovary	IV	501	None	Adeno	Pe	Chemo	883
9	87 F	Melanoma	IV	Simultaneously	Pain, hemorrhage	Melanoma	Lu, Li, Pe, Bo, SI, Ad, S	BSC	112
10	67 F	Pancreas	II B	855	None	N/A	Li, Pe, LN	BSC	98
11	69 M	Bladder	III B	512	None	N/A	Lu, Li, Bo, Br, Pl, Ad, SI, Pe	BSC	69
12	84 F	Kidney	II	5350	Pain, hemorrhage	Clear	Pa, LN	ESD	Alive
13	79 M	DLBCL	I	1028	Pain	N/A	Li, Pe, LN	BSC	427
14	79 F	DLBCL	IV	Simultaneously	Pain, hemorrhage	DLBCL	Lu, Ad	BSC	133
15	81 F	Breast	IV	456	None	N/A	Lu, Li, Bo, Pl, Pe	BSC	17

Clinical findings

Symptoms associated with gastric metastasis were nonspecific. During gastric metastasis diagnosis, nine patients were asymptomatic, six had abdominal pain, and three had bleeding. Five patients were diagnosed with primary cancer and gastric metastasis simultaneously. Out of the 15 patients, 13 already had metastases to multiple organs during the diagnosis of gastric metastasis. The colon was the most common site of primary cancer (n=3). Other sites such as lung, diffuse large B-cell lymphoma (DLBCL), and pancreas accounted for two patients each, and the malignant melanoma, kidney, breast, esophagus, ovary, and bladder had one patient each. During the diagnosis of primary cancer, majority of the patients were already at stage IV (n=10); meanwhile, stages III, II, and I accounted for one, three, and one patients, respectively. This clinical staging was based on the TNM classification of the American Joint Committee on Cancer [8]. The pathological diagnosis of gastric metastasis was confirmed in 10 patients.

CT and endoscopic findings of gastric metastasis

**Figure 2:**
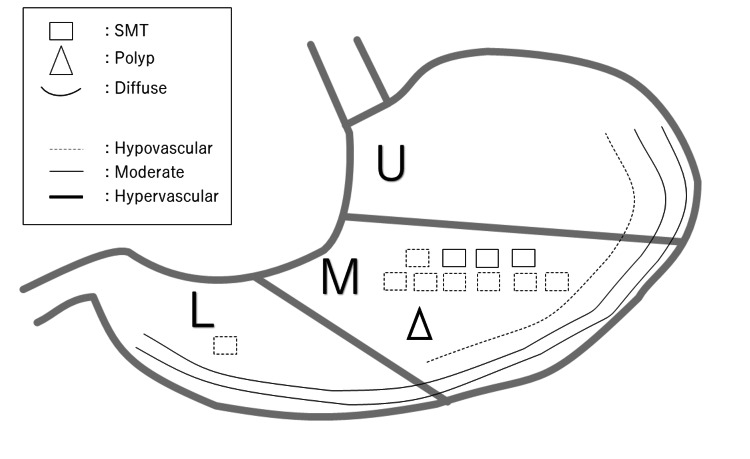
Endoscopic and CT findings in schema.

Table 2 and Figure 2 show the CT and endoscopic findings. On CT images, the median tumor diameter was 18 (12-135 mm). Tumors were mainly located in the middle third of the stomach (n=11, 73.3%), followed by the upper to the lower stomach (diffuse pattern) in two (13.3%) patients, lower third stomach in one (6.7%) patient, and the upper to the middle of the stomach in one (6.7%) patient.

**Table 2:** Endoscopic finding and CT finding. *Note*: SMT, submucosal tumor; N/A, not available; U, upper; M, middle; L, lower; hypo, hypovascular; hyper, hypervascular

No.	Endoscopic findings	Ulcer/erosion on endoscopy	Location/shape/size (mm) on CT	Contrast enhancement pattern
1	SMT	None	L/SMT/15	Hypo
2	SMT	Ulcer	M/SMT/19	Hypo
3	SMT	Ulcer	M/SMT/20	Moderate
4	SMT	Ulcer	M/SMT/15	Hypo
5	SMT	Ulcer	M/SMT/27	Hypo
6	SMT	None	M/SMT/17	Hypo
7	SMT	None	M/SMT/17	Moderate
8	SMT	None	M/SMT/48	Hypo
9	SMT	Ulcer	M/SMT/12	Hypo
10	N/A	N/A	M/SMT/16	Hypo
11	N/A	N/A	M/SMT/15	Moderate
12	Polyp	Erosion	M/polyp/100	Hyper
13	Diffuse	Ulcer	UML/diffuse/135	Moderate
14	Diffuse elevated lesion	Erosion	UM/diffuse/70	Hypo
15	N/A	N/A	UML/diffuse/unmeasurable	Moderate

Regarding tumor shape, CT detected an SMT pattern in most patients (n=11, 73.3%), followed by a diffuse wall thickness pattern in three (20.0%) patients, and a polypoid pattern in one (6.7%) patient. The contrast enhancement of the tumor was hypovascular, hypervascular, and moderate in nine (60%), one (6.7%), and five (33.3%) patients, respectively.

Moreover, endoscopic findings showed an SMT pattern in nine (60%) patients, diffuse lesions in two (13.3%) patients, and a polyp pattern in one (6.7%) patient. Three patients did not undergo endoscopy. Endoscopic findings in patients who underwent endoscopy were consistent with CT findings. Additionally, six (33.3%) patients had gastric tumor ulcers, and two (13.3%) had tumor erosion.

Treatment options and clinical outcomes

**Figure 3:**
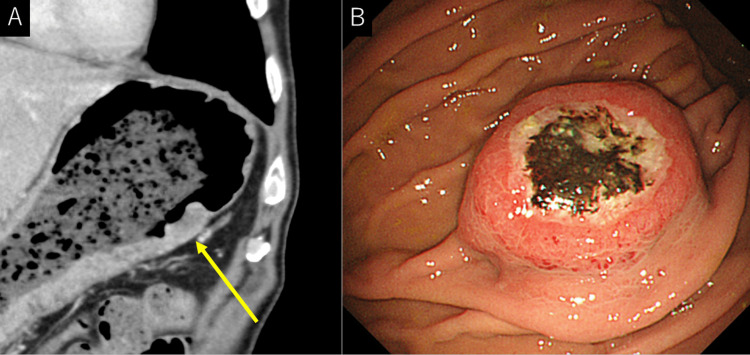
A representative case. A, contrast-enhanced CT, coronal image; B, endoscopy A case of a 65-year-old man suffering from lung cancer with multiple lung and lymph node metastases. CT showed an SMT lesion on the greater curvature of the gastric body (Figure 3A, arrow). Endoscopy revealed a self-destructing SMT at the top of the body of the stomach (Figure 3B). SMT, submucosal tumor

As for the treatment course, the majority of the patients received BSC (n=7); surgery, chemotherapy, and endoscopic treatment were provided to three, three, and two patients, respectively. The median survival time from the date of gastric metastasis diagnosis was 112 (17-883) days. Only one patient, who had renal cell carcinoma (RCC), remained alive after endoscopic treatment. No recurrence of abdominal symptoms or melena was seen at five years of follow-up. However, we could not identify the outcomes of the three patients. Figure 3 shows the representative case.

## Discussion

This study reviewed the CT imaging findings of 15 patients with gastric metastases diagnosed at our institution. The symptoms associated with gastric metastasis were nonspecific. The SMT pattern (73.3%) in the middle third of the stomach (73.3%) was the most recognized CT image pattern of gastric metastasis. The median tumor diameter was 18 mm. This CT finding was similar to the endoscopic finding. As for the treatment course, 47% of patients received BSC, while others chose surgery, chemotherapy, and endoscopic treatment. The median survival time from the date of gastric metastasis diagnosis was 112 days.

In previous reports of endoscopic findings, gastric metastases often showed gastric cancer-like lesions or SMT patterns [3, 9-12]. In the present study, gastric metastases most frequently showed SMT patterns (11 cases), and lesions with ulceration were seen in five cases. Many of these similar lesions could have been recognized as gastric cancer-like lesions in other studies. The reason why SMT shapes are often seen in gastric metastases may be related to the abundant blood flow in the stomach. The mechanism of gastric metastasis is still vaguely understood, but it may include hematogenous, lymphatic, peritoneal dissemination, and direct tumor invasion. In hematogenous metastasis, circulating cancer cells may be trapped in the submucosa of the stomach and develop as SMT [3, 9]. In addition, the micro lymphatic system in the submucosa of the esophagus is thought to be connected to the micro lymphatic system in the submucosa of the stomach; this connection may be related to the mechanism of gastric metastasis [9]. However, we consider that gastric metastases with SMT patterns are difficult to differentiate on CT imaging alone from primary gastric tumors such as gastrointestinal stromal tumors (GISTs), leiomyomas, etc. If a new gastric SMT appears with cancer progression and the enhancement pattern is consistent with the primary cancer site, gastric metastasis may be diagnosed. In our patient with esophageal cancer, gastric metastasis also took the form of an SMT. Regarding breast cancer, gastric metastasis usually originates from lobular carcinoma rather than ductal carcinoma. In addition, endoscopy for patients with breast cancer shows mainly diffuse linitis plastica-like infiltration [13]. The diffuse invasion was also found in our patient with gastric metastasis of breast cancer. Although the mechanism of this phenomenon has not been elucidated, it may be a specific finding.

In several reports on endoscopic findings, gastric metastases were often located in the middle or upper third of the stomach [2, 10-12]. In this study, gastric metastasis was also most common in the middle third of the stomach. This finding also suggests an association with the blood flow in the stomach. Hematogenous metastases may be more prevalent in the middle third of the stomach because of the hemodynamics of the stomach, which receives blood flow from multiple directions.

In this study, gastric metastases often showed SMT patterns in the middle third of the stomach on CT. Although the detailed mechanism is unknown, it was expected that the blood flow control of the stomach is involved. The possibility of gastric metastasis may need to be considered, especially when the above findings are seen on CT in cancer-bearing patients. Early intervention may reduce the disadvantages to the patient, such as hemorrhagic events.

According to a previous report that analyzed the clinical characteristics and examined the prognosis of gastrointestinal metastasis of lung cancer [6], the mean time from the diagnosis of gastric metastases to death is 100 days. The prognosis for this study was longer than the previous report. The wide range of prognosis (17-883 days) and the variety of primary lesions in this study might have an effect on the prognosis of this study.

The follow-up of malignant tumors with CT is one of the most used methods. Early detection of gastric metastases with CT would improve the patient's quality of life. It would also predict prognosis and have a significant impact on the response. Therefore, it is important to evaluate the features of CT findings.

This study has some limitations. It is a retrospective, single-center study, has a small sample size, and included some patients with no endoscopic or pathological findings. Therefore, future studies should collect a large number of cases at multiple centers and positively consider the clinical course, treatment, and prognosis.

## Conclusions

In this study, two radiologists reviewed the characteristics of CT imaging findings of gastric metastases in 15 cases experienced at our hospital. As a result, in many cases, lesions were found in the middle third of stomach, and the shape was SMT pattern. This may be a CT imaging finding characteristic of gastric metastasis. This was also reported in a study on the findings of gastric metastases on endoscopy. CT is widely used in the follow-up of cancer patients, and we believe that it is necessary to consider gastric metastasis when the above findings are observed. This study was conducted at a single institution and the number of cases is small, further studies with more cases are needed to confirm our findings.
